# Heartbeat Detection in Gyrocardiography Signals without Concurrent ECG Tracings

**DOI:** 10.3390/s23136200

**Published:** 2023-07-06

**Authors:** Salvatore Parlato, Jessica Centracchio, Daniele Esposito, Paolo Bifulco, Emilio Andreozzi

**Affiliations:** Department of Electrical Engineering and Information Technologies, University of Naples Federico II, Via Claudio 21, 80125 Naples, Italy; sal.parlato@studenti.unina.it (S.P.); daniele.esposito@unina.it (D.E.); emilio.andreozzi@unina.it (E.A.)

**Keywords:** gyrocardiography, seismocardiography, heartbeat detection, template matching, heart rate, mechanocardiography

## Abstract

A heartbeat generates tiny mechanical vibrations, mainly due to the opening and closing of heart valves. These vibrations can be recorded by accelerometers and gyroscopes applied on a subject’s chest. In particular, the local 3D linear accelerations and 3D angular velocities of the chest wall are referred to as seismocardiograms (SCG) and gyrocardiograms (GCG), respectively. These signals usually exhibit a low signal-to-noise ratio, as well as non-negligible amplitude and morphological changes due to changes in posture and the sensors’ location, respiratory activity, as well as other sources of intra-subject and inter-subject variability. These factors make heartbeat detection a complex task; therefore, a reference electrocardiogram (ECG) lead is usually acquired in SCG and GCG studies to ensure correct localization of heartbeats. Recently, a template matching technique based on cross correlation has proven to be particularly effective in recognizing individual heartbeats in SCG signals. This study aims to verify the performance of this technique when applied on GCG signals. Tests were conducted on a public database consisting of SCG, GCG, and ECG signals recorded synchronously on 100 patients with valvular heart diseases. The results show that the template matching technique identified heartbeats in GCG signals with a sensitivity and positive predictive value (PPV) of 87% and 92%, respectively. Regression, correlation, and Bland–Altman analyses carried out on inter-beat intervals obtained from GCG and ECG (assumed as reference) reported a slope of 0.995, an intercept of 4.06 ms (R^2^ > 0.99), a Pearson’s correlation coefficient of 0.9993, and limits of agreement of about ±13 ms with a negligible bias. A comparison with the results of a previous study obtained on SCG signals from the same database revealed that GCG enabled effective cardiac monitoring in significantly more patients than SCG (95 vs. 77). This result suggests that GCG could ensure more robust and reliable cardiac monitoring in patients with heart diseases with respect to SCG.

## 1. Introduction

Heart rate monitoring plays a key role in the assessment of cardiovascular function. Continuous, long-term cardiac monitoring has long been recognized as a critical task to improve the diagnosis and treatment of cardiac diseases [1,2,3,4]. To date, electrocardiography (ECG) is widely recognized as the gold standard for heart rate measurement via recording the myocardial electrical activity. However, ECG examinations can only be performed by skilled clinical professionals using approved medical devices. In addition, various practical drawbacks, such as detachments/slipping of electrodes, drying of electrolytic gel, and susceptibility to electromagnetic interferences, make electrocardiography not appealing for continuous, long-term monitoring, particularly in non-clinical settings [5,6,7,8,9,10,11]. Heart rate monitors based on plethysmographic techniques are available as alternatives to ECG, as well as in the form of wearable devices (e.g., smartwatches and fitbands). However, their strong sensitivity to motion artifacts usually limits their application to continuous, long-term monitoring for clinical purposes [11,12,13,14,15,16,17,18]. Cardio-mechanical monitoring techniques represent a further alternative for non-invasive assessment of cardiac function. Many of these techniques record the mechanical activity of the heart from a subject’s chest, essentially replicating the ancient practice of palpation [11,19]. From the end of the 19th century, various techniques have been proposed to record the tiny vibrations induced on the human body by the mechanical activity of the cardiovascular system [20]. Indeed, specific events of the cardiac cycle could be monitored by analyzing the peaks and valleys of cardio-mechanical signals. However, the bulky instrumentation and complexity of signal interpretation, as well as the considerable inter-subject variability, led most of these techniques to fall into disuse in favor of the emerging ultrasound imaging, which offered the unprecedented opportunity to visually inspect the anatomy and physiology of the cardiovascular system. Indeed, ultrasound imaging and Doppler techniques are currently reference clinical examinations for assessing the mechanical properties of the myocardium, which cannot be evaluated from ECG. However, ultrasound techniques also require highly qualified specialists, and are not suitable for continuous, long-term cardiac monitoring.

In the last two decades, the manufacturing of small, lightweight, inexpensive sensors based on micro-electromechanical system (MEMS) technologies has rejuvenated the interest in Ballistocardiography (BCG), which records the mechanical vibrations of the whole body due to blood ejection towards the feet [21,22,23,24,25,26], as well as Seismocardiography (SCG), which measures the infrasonic three-dimensional accelerations of the precordium induced by heart contractions [26,27,28,29,30,31,32,33]. In addition, the availability of modern electronic stethoscopes fostered the use of phonocardiography (PCG) to record heart sounds [34,35,36,37,38,39].

Recently, novel, non-invasive, cardio-mechanical monitoring techniques have been introduced, namely Forcecardiography (FCG) [40,41,42,43,44,45,46] and Gyrocardiography (GCG) [47,48,49,50], and even the combination of SCG with BCG [51] and GCG [52,53] has been investigated. FCG captures precordial vibrations using broadband force sensors, which are able to monitor respiration, infrasonic cardiac vibrations, and heart sounds, all simultaneously from a single site on the chest. On the other hand, GCG makes use of tri-axial gyroscopes to measure the infrasonic angular velocities of the thorax induced by heart contraction. Indeed, up to 60% of cardiac vibrational energy has been reported to be contained in the GCG signal [49]. Furthermore, peaks and valleys of GCG signals also have a clear correlation with important cardiac cycle events, thus allowing for the estimation of cardiac time intervals of diagnostic relevance [49,50]. Generally, the angular velocities around the latero–lateral axis (GCGx) and cranio–caudal axis (GCGy) are analyzed. During the cardiac cycle, the twisting and untwisting motion of the heart, which results from the contraction and relaxation of the helically oriented myocardial fibers, causes the chest wall to move accordingly. The specific arrangement of myofibrils, which form two oppositely oriented eight-shaped configurations, is responsible for the torsional motion of the heart. Indeed, during systole, the apex of the heart rotates counterclockwise, while the base rotates clockwise; on the contrary, during diastole, the opposite occurs [54,55,56]. This movement is mainly captured by the *y* component of the GCG signal [49].

Beat-by-beat heart rate monitoring from GCG signals is feasible. Specifically, precise identification of the temporal locations of all heartbeats and accurate measurement of inter-beat intervals are demanded to accomplish this task. Generally, heartbeats are localized by taking advantage of the a priori knowledge of R-peaks on a simultaneous ECG recording [49,57,58,59]. However, GCG-based heartbeat detectors that remove the need for concurrent ECG signals have also been proposed. In this regard, various approaches are available, such as short-term autocorrelation [60,61]; envelope extraction through wavelet [62] or Hilbert [63,64,65] transform, even combined with adaptative thresholding [66], clustering [67] or autoregressive models [68]; calculation of the kinetic energy waveform followed by Sparse Fast Fourier Transform [69]; and the autocorrelated differential algorithm [51,70]. A great part of these methods is based on rather complex algorithms and has only been tested on GCG signals recorded from subjects without any history of cardiovascular diseases. On the contrary, the method presented in [67], which combines GCG envelope extraction and unsupervised k-means clustering, was also assessed on a limited cohort of 12 patients with coronary artery disease. Moreover, GCG signals from 40 patients with atrial fibrillation, 21 patients with coronary artery disease, and 21 patients with myocardial ischemia were considered in [61], but any information is reported on the performance of heartbeat detection and the accuracy of inter-beat interval measurements. Heartbeats have also been recognized by combining GCG and SCG recordings [51,65,71]. As an example, independent component analysis was first employed in [65] for data fusion; then, an envelope-based heartbeat detector was tested on 19 patients with coronary artery disease. Furthermore, in [69], heartbeats were located on the kinetic energy waveform, which was obtained as the ensemble averaging of SCG and GCG signals. Deep learning methods for biosignal analysis [72,73,74] have also been proposed for ECG-free heartbeat detection in SCG [75] and BCG [76] signals, but not in GCG signals.

This study aims to demonstrate the suitability of a template matching approach for ECG-free heartbeat detection in GCG signals. The algorithm proposed here is based on the well-established normalized cross-correlation (NCC) function [77,78,79] to assess the morphological similarity between a selected template and signal chunks. This allows for capturing the heartbeat pattern in the signal without any a priori assumptions about the signal waveform. The performance of the proposed approach was extensively evaluated on SCG signals of 100 patients with one or more valvular heart diseases (VHDs) [80], made available in a public database [81]. To the best of our knowledge, for the first time in the literature, a template matching algorithm has been shown to provide high precision in heartbeat detection, as well as high accuracy in inter-beat intervals estimation, on a large cohort of pathological subjects [80]. As the database considered also contained simultaneous GCG recordings, the feasibility of this method on GCG signals from the same 100 VHD patients was evaluated in this work. To this end, the temporal locations of heartbeats were identified from both GCG signals and reference ECG recordings, and inter-beat interval measurements were compared via statistical analyses. The results showed that even on GCG signals the template matching offers a simple, yet effective and robust way for continuous heart rate monitoring.

## 2. Materials and Methods

### 2.1. Dataset

GCG and ECG signals of 100 VHD patients (59 males and 41 females, age 68 ± 14 years) from a public database [81] were considered in this study. The subjects suffered from one or more VHDs at a moderate or severe stage. In particular, 23 subjects were affected by mitral valve stenosis, 51 by mitral valve regurgitation, 39 by aortic valve stenosis, 28 by aortic valve regurgitation, 40 by tricuspid valve regurgitation, and 59 subjects suffered from more than one VHD (more detailed information on the patients is available in [81] and at the database repository). GCG and ECG signals were recorded on subjects’ chest by means of a tri-axial MEMS gyroscope and an ECG front-end, respectively, integrated in the Shimmer 3 ECG module (Shimmer Sensing, Dublin, Ireland). Signals were acquired simultaneously at a sampling rate of 256 Hz (patient IDs #CP-01 to #CP-70 and #UP-01 to #UP-21) or 512 Hz (patients IDs #UP-22 to #UP-30), from subjects lying supine and breathing at a natural pace. Only GCGy signals and ECG leads II were considered for the analysis. Recordings corresponding to patient ID #UP-28 were discarded due to the lack of a simultaneous ECG signal. In addition, four recordings (patient IDs #CP-24, #CP-31, #CP-55, and #UP-22) were excluded from the analysis because of poor GCG signal quality. Therefore, signals from a total of 95 subjects were analyzed in this study. 

### 2.2. Signals Pre-Processing

**Figure 1:**
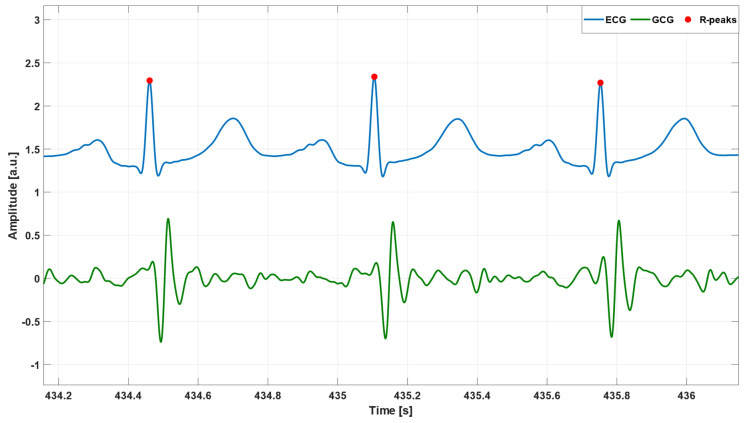
An example of pre-processed ECG (blue line) and GCG (green line) signals from patient #CP-63. Red points mark R peak locations.

Matlab^®^ R2018b (The MathWorks, Inc., 1 Apple Hill Drive, Natick, MA 01760, USA) was used for all signal processing operations. First of all, a linear interpolation was carried out via the Matlab^®^ function interp1 to oversample the signals up to 1 kHz, in order to improve their temporal resolution. Afterwards, the ECG signal was filtered in the 0.5–40 Hz frequency band using a fourth order zero-lag Butterworth band-pass filter; then, the 50 Hz powerline interference and its higher harmonics were filtered out by means of a comb notch filter; finally, the temporal locations of R-peaks were identified by the well-kwown Pan and Tompkins algorithm [82], implemented in the BioSigKit Matlab^®^ toolbox [83]. On the other hand, the GCG signal was processed via a fourth order zero-lag Butterworth filter with 7–30 Hz pass band. Figure 1 shows an example of pre-processed ECG and GCG signals from patient #CP-63. 

### 2.3. Template Matching

A template matching algorithm, previously proposed in a SCG [80] study, was implemented for heartbeat localization in GCG signals. As already reported in [80], the template matching first requires selecting a heartbeat template within the GCG signal, then calculating the NCC function between the selected template and GCG signal chunks, and finally identifying the temporal locations of the NCC peaks. 

#### 2.3.1. Template Selection

**Figure 2:**
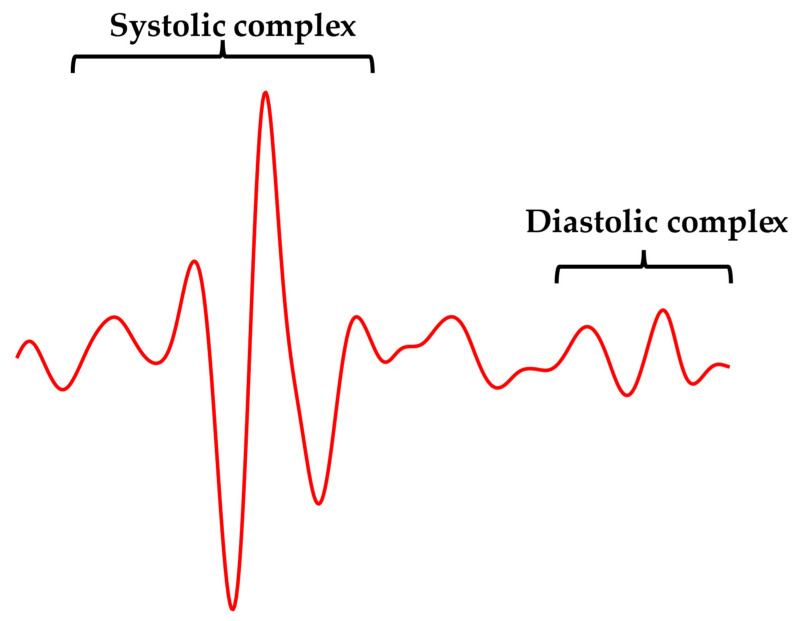
An example of the recommended template waveform extracted from subject #CP-63.

**Figure 3:**
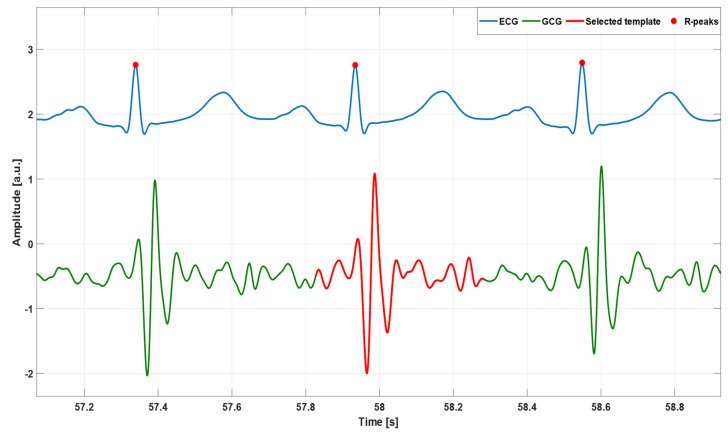
An example of an ECG signal (blue line), GCG signal (green line), and selected template (red line) from subject #CP-63. The template corresponds to that highlighted in Figure 2. Red points mark R-peak locations.

First, a template was identified in each GCG signal in order to capture the typical heartbeat waveform, which was regarded as a morphological reference. As illustrated in Figure 2, the template was selected according to the following criterion: “the *template* should start two or three waves before the highest local maximum in the systolic complex, where the amplitude of the waves is significantly lower than systolic peak magnitude, and should end after the last wave in the diastolic complex”. All GCG signals included in the dataset met this criterion. Hence, a template comprising both the systolic and diastolic complexes of the selected cardiac cycle was chosen for all of the GCG signals analyzed. Figure 3 shows an example of template selection for subject #CP-63.

#### 2.3.2. Heartbeats Localization on Normalized Cross-Correlation Function

**Figure 4:**
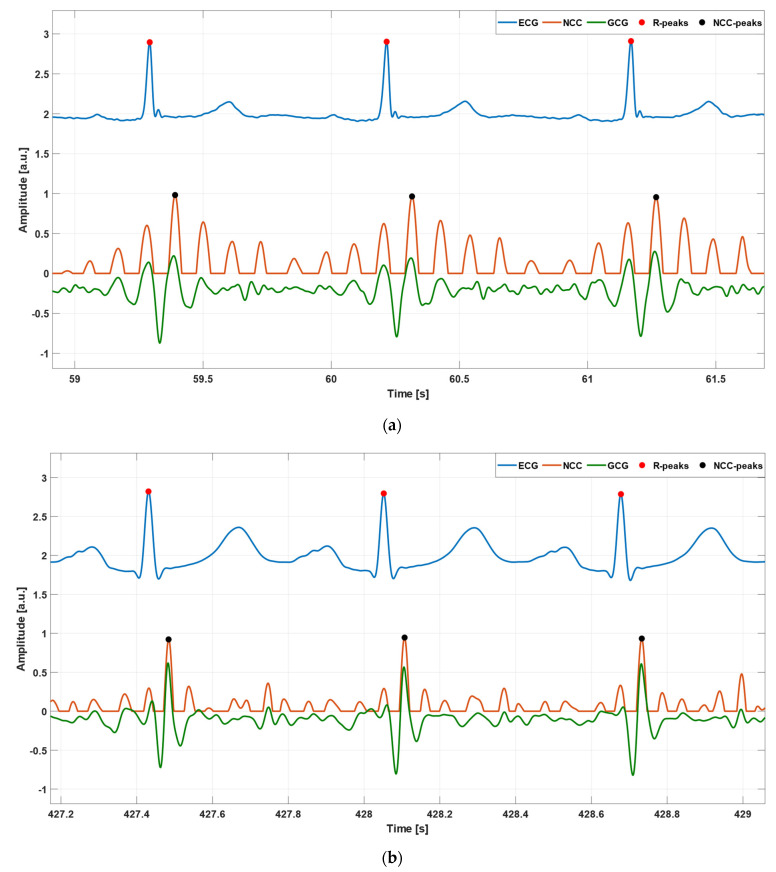
Some excerpts of ECG (blue line), GCG (green line), and NCC (orange line) signals from subjects: (**a**) #CP-08 and (**b**) #CP-63. Red and black points mark the temporal locations of R peaks and NCC peaks, respectively.

The NCC function was considered as a similarity measure between the template and signal chunks to find those that most resemble the template, as in [80]. Local maxima in the NCC function corresponded to signal chunks with highest local similarity to the selected template, so they marked the temporal locations of heartbeats. Therefore, once a template was selected, the NCC function was computed between the template and GCG signal chunks, after local mean removal. Then, the local maxima were identified in the NCC function obtained via the Matlab^®^ function findpeaks, by setting a minimum peak prominence of 0.5 and a minimum peak distance of 500 ms for all of the GCG signals included in the dataset. Figure 4 shows some examples of heartbeat detection in the NCC function for patients #CP-63 and #CP-08.

#### 2.3.3. Inter-Beat Intervals Estimation

**Figure 5:**
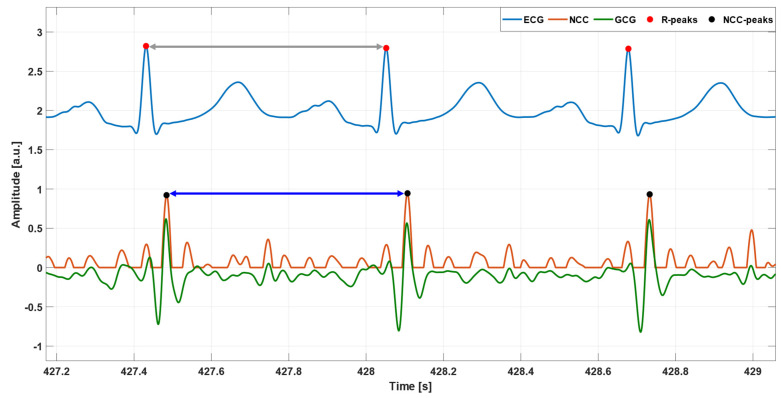
An example of inter-beat interval estimation from subject #CP-63 on ECG (blue line) and NCC (orange line) signals. For the ECG signal, the inter-beat interval was estimated as the temporal difference between two consecutive R-peaks (grey double arrow). For the NCC signal, the inter-beat interval measurement was obtained as the temporal difference between two consecutive NCC peaks (electric blue double arrow).

Once NCC peak detection was completed, wrong and missed heartbeat detections among all NCC peaks were annotated with the support of reference ECG signals, as reported in [80]. Multiple NCC peaks within one cardiac cycle were considered as false positives (FPs); single NCC peaks within one cardiac cycle occurring at temporal locations that were unlikely to correspond to matched heartbeat templates were considered as detection errors (DEs); cardiac cycles with no NCC peaks were considered as false negatives (FNs). DEs essentially marked cardiac cycles where only one FP was found, and no true heartbeat was detected, so they contributed both to the number of FPs and FNs. Finally, inter-beat intervals were estimated from ECG and GCG signals as temporal differences between consecutive R-peaks and NCC-peaks, respectively, as depicted in Figure 5. Inter-beat intervals corrupted by FNs and DEs were obviously removed from the analysis.

### 2.4. Statistical Analyses

Sensitivity and positive predictive value (*PPV*) were considered as performance evaluation metrics of heartbeat detection. These metrics are defined by the following mathematical expressions:

(1) $$Sensitivity=\frac{TP}{TP+FN+DE}\cdot 100,$$

(2) $$PPV=\frac{TP}{TP+FP+DE}\cdot 100,$$

where *TP*, *FN*, *FP,* and *DE* indicate the number of true positives, false negatives, false positives, and detection errors, respectively.

Furthermore, regression, correlation, and Bland–Altman [84,85] analyses were carried out via the Matlab^®^ function bland–altman-and-correlation-plot [86], to compare the inter-beat interval measurements obtained from GCG signals with those provided by the reference ECG signals. In addition, the results obtained from the analysis of GCG signals were compared with those reported in the previous work on SCG signals [80], to assess the performance of the proposed template matching algorithm in the estimation of inter-beat intervals from the GCG and SCG signals acquired on the same VHD patients.

## 3. Results

**Figure 6:**
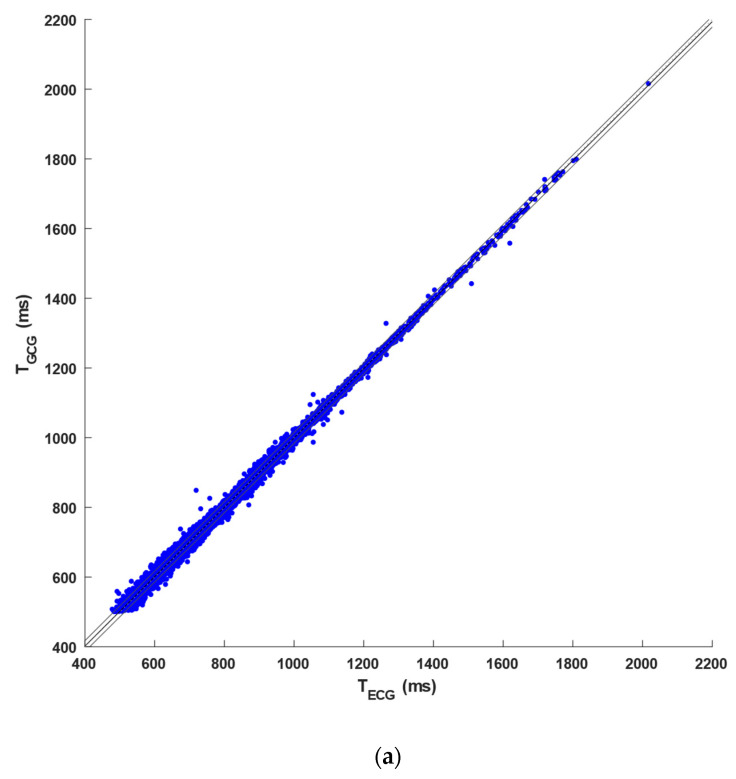

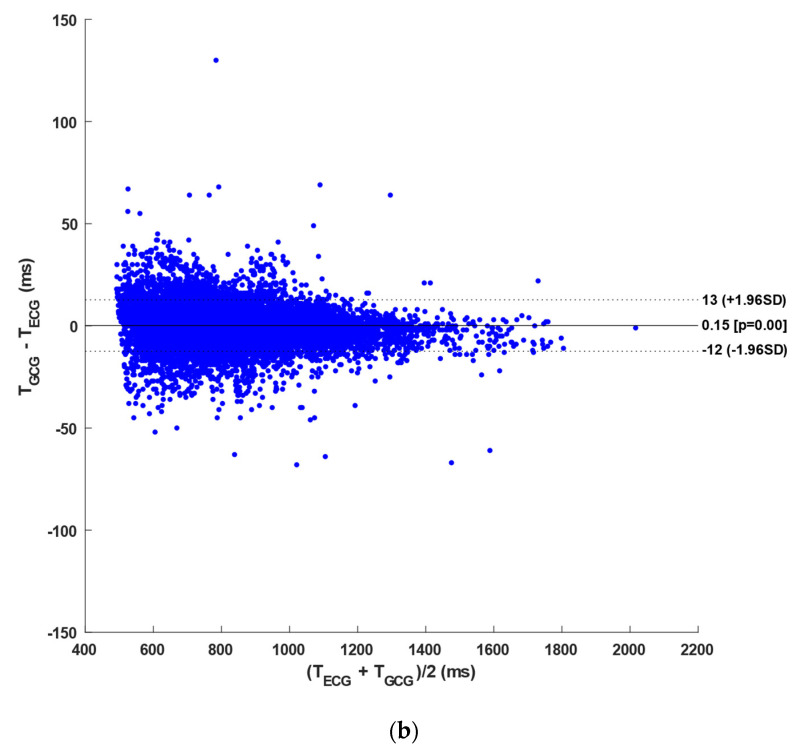
Statistical analyses on inter-beat intervals obtained from GCG signals of the 95 VHD patients included in the dataset: (**a**) results of the regression and correlation analysis and (**b**) results of the Bland–Altman analysis.

The number of ECG heartbeats (R-peaks) and GCG heartbeats (NCC-peaks) identified for each subject, together with the number of FPs, FNs, and DEs, are outlined in Table A1 in Appendix A. This table also reports the number of inter-beat intervals compared via the statistical analyses. An overall number of 40,527 and 38,565 heartbeats were identified in the ECG and GCG signals, respectively. In particular, 35,383 TPs, 526 FPs, 2486 FNs, and 2656 DEs were detected in the GCG signals. Hence, heartbeat detection in GCG signals achieved a sensitivity of 87% and a PPV of 92%. Furthermore, an overall number of 31,831 inter-beat intervals were compared via regression, correlation, and Bland–Altman analyses. The regression and correlation analysis reported a slope of 0.995, an intercept of 4.06 ms (R^2^ > 0.99), and a Pearson’s correlation coefficient *r* of 0.9993 (*p* < 0.001), as illustrated in Figure 6a. Moreover, limits of agreement (LoA) of [−12.85, 13.15] ms, with a 95% confidence interval (95% CI) of [−12.97, 13.27] ms, and a negligible bias of 0.15 ms (*p* < 0.001), with a 95% CI of [0.08, 0.22] ms, were obtained from the Bland–Altman analysis (see Figure 6b). The results of statistical analyses are also summarized in Table 1.

**Figure 7:**
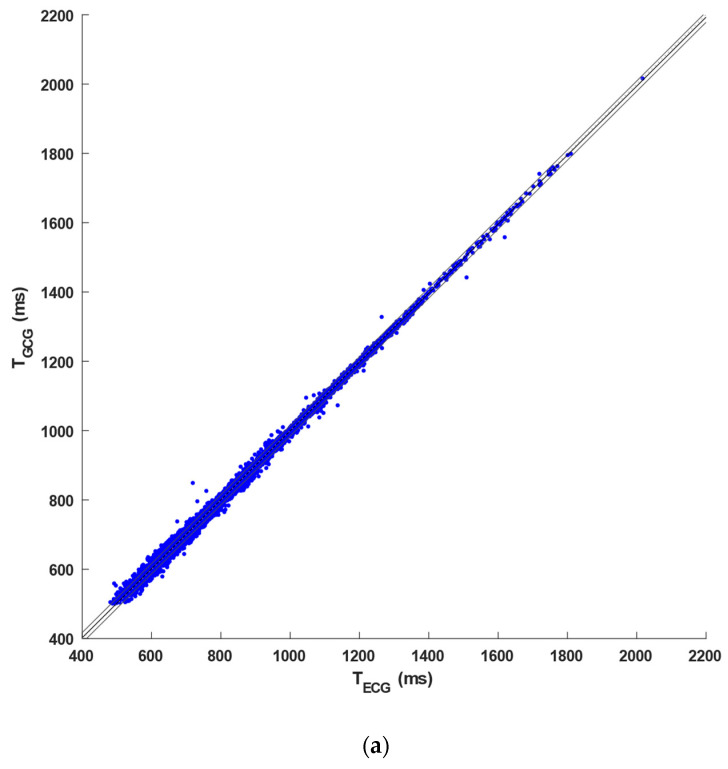

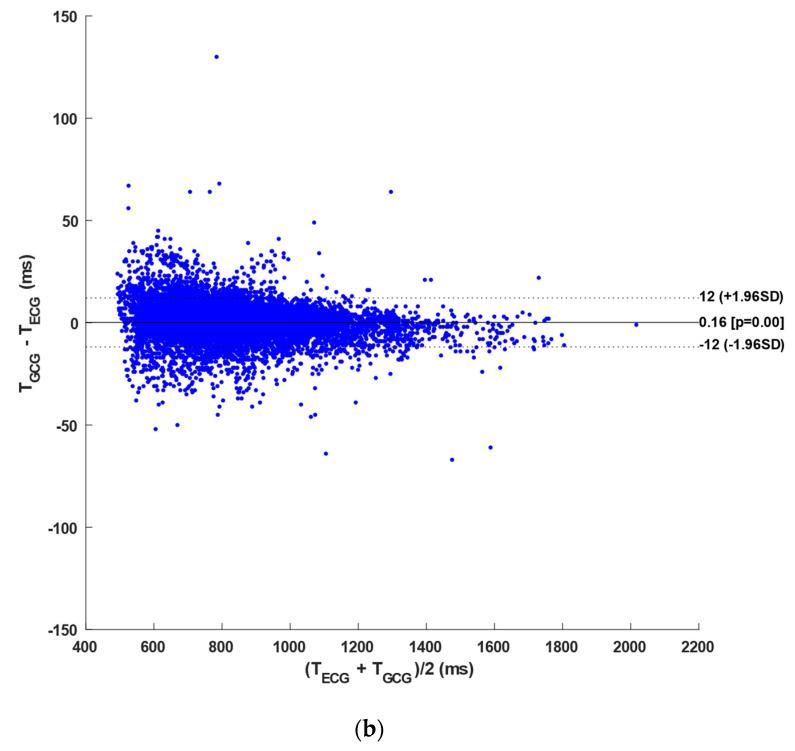
Statistical analyses on inter-beat intervals obtained from GCG signals of the 76 VHD patients: (**a**) results of the regression and correlation analysis and (**b**) results of the Bland–Altman analysis.

To compare the performance of heartbeat detection in GCG and SCG signals from the same database [81], Table A2 in Appendix A reports, for each of the 100 VHD patients, the number of heartbeats identified in the ECG signals; the number of TPs, FPs, and FNs detected in the GCG recordings analyzed in this study; and the number of TPs, FPs, and FNs recognized in the SCG recordings obtained in a previous study [80]. In particular, the table also highlights which patients were excluded from the analyses of GCG and/or SCG signals due to very poor signal quality (see dashes in the table). In addition, a comparison of the performances achieved in the inter-beat interval estimation from GCG and SCG recordings against the reference ECG was carried out. To this aim, 76 patients were considered, for which both GCG and SCG analysis was feasible, and then regression, correlation, and Bland–Altman analyses of inter-beat intervals were performed. In detail, a total of 41,320 heartbeats were detected in the ECG signals, while 35,383 TPs, 3182 FPs, and 5142 FNs were identified in GCG recordings, and 29,583 TPs, 2976 FPs, and 3678 FNs were detected in the SCG recordings. Hence, the GCG signals scored a sensitivity of 89% and a PPV of 93%, while SCG scored a sensitivity of 90% and a PPV of 91.5%. Moreover, a slope of 0.995, an intercept of 4.51 ms (R^2^ > 0.99), and a Pearson’s correlation coefficient *r* of 0.9993 (*p* < 0.001) were obtained for GCG signals, while a slope of 0.995, an intercept of 3.92 ms (R^2^ > 0.99), and a Pearson’s correlation coefficient *r* of 0.9993 (*p* < 0.001) were found for the SCG signals (see Figure 7a). As shown in Figure 7b, the Bland–Altman analyses reported a negligible bias of 0.16 ms (*p* < 0.001), with a 95% CI of [0.09, 0.23] ms, and LoA of [−11.84, 12.16] ms, with a 95% CI of [−11.97, 12.29] ms, for the GCG signals, as well as a non-significant bias (*p* = 0.09), with 95% CI of [0.01, 0.17] ms, and LoA of [−12.91, 13.09] ms, with 95% CI of [−13.05, 13.23] ms, for the SCG signals. The results of these statistical analyses are also outlined in Table 2 (see [80] for further details on the results obtained for SCG signals).

## 4. Discussion

The proposed method was able to recognize heartbeats in GCG signals of 95 out of 99 patients (96%) with a sensitivity and PPV of 87% and 92%, respectively, also providing accurate measurements of inter-beat intervals compared with ECG (LoA of about ± 13 ms). It is worth noting that, when applied on SCG, the templatematching method ensured accurate heartbeat detection in 77 out of 99 patients (78%) with similar values of sensitivity and PPV, as well as of LoA for inter-beat interval estimation. In both SCG and GCG, the inability to ensure an acceptable performance in heartbeat detection was due to an extremely poor signal quality.

The measurement errors in inter-beat intervals obtained by SCG and GCG compared with ECG have various contributions. First of all, assuming the ideal capability of locating the timing of the aortic valve opening with maximum accuracy in SCG and GCG signals (AO peaks), it must be considered that the time delay between ECG R peaks and AO peaks, which is a component of the pre-ejection period, changes over time. Therefore, if the R–AO time delays of two consecutive heartbeats are different, then the related AO–AO interval will differ from the R–R interval, thus leading to a measurement error. Moving forward to less ideal conditions, it is important to consider that motion artifacts and noise may corrupt the heartbeat morphology in SCG and GCG signals, thus potentially leading to imprecise localization of the AO peaks, and eventually to errors in inter-beat intervals measurements. Finally, it must be considered that in the proposed approach the heartbeat markers are the peaks of NCC between the selected template and the signals, and not the actual AO peaks. This is important as the time delay between the NCC peaks and the actual AO peaks may change over time, because the heartbeat morphology is not always exactly the same of the template, so the local maximum of NCC may not always occur at a fixed time distance from the related actual AO peak. Again, different AO–NCC-peaks time delays in two consecutive heartbeats would lead to differences between NCC peak–NCC peak intervals and AO–AO intervals, and eventually in additional measurement errors with respect to R–R intervals. Anyway, the impact of these errors on the parameters of clinical relevance, such as the mean heart rate and HRV indices, has yet to be evaluated, and will be the subject of a future study.

This is the first study that assessed the performance of an algorithm for ECG-free heartbeat detection in GCG signals on such a large cohort of subjects. In addition, it is also the first time that the heart monitoring performances of SCG and GCG have been thoroughly compared for such a large cohort of pathological subjects. Indeed, the results suggest that, for patients affected by valvular heart diseases, the heartbeat morphology is more stable in GCG than in SCG signals. This result has important implications in the development of devices and methods for continuous, long-term patient surveillance based on cardio-mechanical monitoring techniques. In fact, the superior performances achieved by GCG suggest that its use could ensure a more reliable and robust monitoring of actual patients. However, considering that electronic microchips of inertial measurement units incorporating both accelerometers and gyroscopes are widely available nowadays, it might be advantageous to apply sensor fusion approaches to design heartbeat detectors that jointly use SCG and GCG signals, in order to leverage their strengths and compensate for their weaknesses. Undoubtedly, the availability of a quantitative index of signal quality could help to dynamically identify the most reliable signal, thus possibly improving the performance of heartbeat detection.

Overall, the proposed templatematching method achieves very high performance on both SCG and GCG signals, which makes it effective for ECG-free cardiac monitoring. The modest computational burden of the proposed method also makes it very efficient and suitable for implementation on resource-constrained computing platforms, possibly also in real time. To date, not many ECG-free methods have been proposed in the literature. The few proposed methods are based on rather complex procedures, or on computationally intensive transformations, or even on machine learning and deep learning techniques, which require very high-performing processors. However, such artificial intelligence methodologies can potentially provide valuable support for the classification and interpretation of signals to offer not only information on heart rhythm, but also on other cardiovascular functions and pathologies.

The results obtained in this study cannot be directly compared with those of previous studies for different reasons. First, the performance of different algorithms must be compared on the same data for consistency, but none of the methods proposed in the literature has been assessed on the same database. Furthermore, other methods have been evaluated on much smaller cohorts of subjects. Data from these cohorts were likely to be characterized by a lower inter-subject variability, which has long been recognized as a factor affecting the accuracy of biosignal analysis. In addition, these cohorts mainly consisted of healthy subjects, sometimes with the presence of a small number of pathological subjects. It is well known that cardio-mechanical signals from pathological subjects exhibit atypical waveforms, with higher morphological variability and difficulty in annotation and interpretation compared with healthy subjects [87], thus making the heartbeat detection task far more complex. Therefore, the methods proposed in the literature have been tested on signals with undoubtedly higher quality and with lower inter-subject variability than those considered in this study, so it is more likely that the results of other methods have been overestimated.

Obviously, the need for manual selection of the heartbeat template is the main limitation for the use of the template-matching method by unskilled users who are unable to follow the suggested guidelines. Therefore, an automated template selection algorithm could be beneficial to improve the reproducibility of the proposed technique.

## 5. Conclusions

This study presents an extensive performance analysis of an ECG-free heartbeat detection method in Gyrocardiography signals. The method is based on a template matching approach and was tested on GCG signals from a public database of 100 patients with valvular heart diseases. The algorithm has previously been tested on Seismocardiography signals from the same database.

This study provides, for the first time, an extensive comparison of heart rate monitoring performances of SCG and GCG on a large cohort of pathological subjects. The morphology of heartbeats turned out to be more stable in GCG than in SCG signals, thus providing a superior performance in heartbeat detection. According to these results, GCG could ensure a more reliable and robust monitoring of actual patients. However, these findings should be confirmed also on patients affected by other cardiac diseases.

Overall, the proposed method achieves very high performance for both SCG and GCG signals, and presents a modest computational burden compared with other methods proposed in the literature. These aspects make the template matching algorithm effective and efficient for ECG-free cardiac monitoring. It is also suitable for real-time implementation on resource-constrained computing platforms, which is appealing to enable the continuous, long-term monitoring of heart rate.

## Figures and Tables

**Table 1:** Results of statistical analyses on the GCG signals of the 95 VHD patients included in the dataset: 95% CI_bias_ is the 95% confidence interval of the bias and 95% CI_LoA_ is the 95% confidence interval of the limits of agreement.

**Sample size**	***N° of subjects***	95
	***N° of compared inter-beat intervals***	31,831
**Performance of heartbeat detection**	***Sensitivity (%)***	87
	***PPV (%)***	92
**Results of regression analysis**	***Slope***	0.995
	***Intercept (ms)***	4.06
	***R^2^***	0.9985
**Results of correlation analysis**	***r***	0.9993 (*p* < 0.001)
**Results of Bland-Altman analysis**	***Bias (ms)***	0.15 (*p* < 0.001)
	***95% CI_bias_ (ms)***	[0.08, 0.22]
	***LoA (ms)***	[−12.85, 13.15]
	***95% CI_LoA_ (ms)***	[−12.97, 13.27]

**Table 2:** Results of statistical analyses performed on GCG and SCG signals from the same 76 VHD patients: 95% CI_bias_ is the 95% confidence interval of the bias and 95% CI_LoA_ is the 95% confidence interval of the limits of agreement.

		GCG	SCG
**Sample size**	***N° of subjects***	76	76
	***N° of compared inter-beat intervals***	26,308	26,913
**Performance of heartbeat detection**	***Sensitivity (%)***	89	90
	***PPV (%)***	93	91.5
**Results of regression analysis**	***Slope***	0.995	0.995
	***Intercept (ms)***	4.51	3.92
	***R^2^***	0.9987	0.9986
**Results of correlation analysis**	***r***	0.9993 (*p* < 0.001)	0.9993 (*p* < 0.001)
**Results of Bland-Altman analysis**	***Bias (ms)***	0.16 (*p* < 0.001)	0.09 (*p* = 0.09)
	***95% CI_bias_ (ms)***	[0.09, 0.23]	[0.01, 0.17]
	***LoA (ms)***	[−11.84, 12.16]	[−12.91, 13.09]
	***95% CI_LoA_ (ms)***	[−11.97, 12.29]	[−13.05, 13.23]

## Data Availability

All of the relevant research data will be made available upon request after the publication of the paper.

## Appendix A

**Table A1:** Number of heartbeats detected in the ECG and GCG signals, along with the number of FP, FN, and DE identified in GCG signals and the number of compared inter-beat intervals.

Patient ID #	ECG Heartbeats (R-Peaks)	GCG Heartbeats (NCC-Peaks)	FP	FN	DE	Compared Inter-Beat Intervals
CP-01	448	448	0	0	2	443
CP-02	651	633	3	21	3	609
CP-03	481	474	2	9	9	451
CP-04	661	629	2	34	16	562
CP-05	509	502	2	9	6	484
CP-06	294	286	2	10	7	264
CP-07	451	450	3	4	5	435
CP-08	544	544	0	0	4	536
CP-09	364	320	2	46	57	190
CP-10	506	485	19	40	41	347
CP-11	656	627	3	32	57	500
CP-12	423	425	9	7	3	404
CP-13	837	829	0	8	1	819
CP-14	472	429	6	48	27	319
CP-15	630	618	0	12	16	590
CP-16	355	354	1	2	4	342
CP-17	362	366	13	9	42	272
CP-18	601	599	1	3	1	593
CP-19	484	473	8	19	32	404
CP-20	391	393	3	1	0	388
CP-21	247	247	0	0	2	242
CP-22	610	534	5	81	71	368
CP-23	235	235	0	0	0	234
CP-25	602	494	10	118	147	166
CP-26	389	379	2	12	6	352
CP-27	130	130	1	1	0	127
CP-28	238	239	1	0	2	233
CP-29	290	192	0	98	20	91
CP-30	523	512	0	11	3	500
CP-32	527	493	15	49	91	298
CP-33	449	487	40	2	10	424
CP-34	462	455	2	9	5	436
CP-35	484	483	13	14	125	269
CP-36	386	409	26	3	197	73
CP-37	342	343	3	2	3	331
CP-38	406	339	23	90	53	161
CP-39	518	515	0	3	39	448
CP-40	509	413	0	96	19	328
CP-41	349	349	0	0	1	346
CP-42	346	343	6	9	18	296
CP-43	460	408	15	67	138	159
CP-44	321	321	0	0	4	312
CP-45	357	359	18	16	58	232
CP-46	499	489	3	13	21	441
CP-47	537	457	11	91	38	319
CP-48	637	588	33	82	121	313
CP-49	451	425	13	39	27	325
CP-50	781	679	0	102	30	547
CP-51	621	621	0	0	52	543
CP-52	728	494	0	234	54	212
CP-53	562	497	0	65	49	377
CP-54	397	366	1	32	36	275
CP-56	742	629	0	113	6	511
CP-57	507	507	1	1	3	499
CP-58	525	525	1	1	1	521
CP-59	405	405	1	1	1	400
CP-60	512	503	0	9	10	484
CP-61	397	397	0	0	2	393
CP-62	572	500	1	73	5	424
CP-63	610	609	0	1	0	607
CP-64	382	396	14	0	4	373
CP-65	369	369	0	0	5	361
CP-66	468	468	1	1	0	465
CP-67	539	514	10	35	25	429
CP-68	327	348	23	2	1	320
CP-69	587	585	0	2	2	578
CP-70	422	429	19	12	44	324
UP-01	239	232	22	29	58	111
UP-02	286	268	5	23	66	134
UP-03	264	218	1	47	34	135
UP-04	258	247	7	18	23	183
UP-05	462	412	2	52	122	178
UP-06	458	398	0	60	16	325
UP-07	376	372	1	5	3	359
UP-08	257	257	0	0	2	252
UP-09	286	285	2	3	7	267
UP-10	165	161	1	5	12	131
UP-11	417	386	25	56	10	297
UP-12	339	336	2	5	25	283
UP-13	106	104	0	2	7	89
UP-14	340	341	1	0	9	321
UP-15	214	215	1	0	0	213
UP-16	221	220	4	4	36	150
UP-17	613	544	1	70	62	421
UP-18	350	351	1	0	3	343
UP-19	116	116	11	11	45	26
UP-20	617	494	3	126	55	329
UP-21	305	308	3	0	2	300
UP-23	565	570	5	0	3	558
UP-24	349	335	0	14	11	308
UP-25	244	222	12	34	44	131
UP-26	160	158	0	2	1	154
UP-27	269	276	22	15	74	131
UP-29	146	146	1	1	5	133
UP-30	228	230	2	0	39	150
**Total**	**40,527**	**38,565**	**526**	**2486**	**2656**	**31,831**

**Table A2:** Comparison of SCG and GCG results on the whole database. The table reports the number of heartbeats detected in the ECG signals, together with the number of TP, FP, and FN detected in both SCG and GCG recordings (the number of DE was added to those of FP and FN). Black dashes indicate the SCG and/or GCG recordings discarded from the analysis due to very poor signal quality. Recordings of patient #UP-28 were not analyzed because ECG was unavailable (NA: not available).

Patient ID #	ECG Heartbeats (R-Peaks)	SCG			GCG		
		TP	FP	FN	TP	FP	FN
CP-01	448	448	0	0	446	2	2
CP-02	651	606	37	45	627	6	24
CP-03	481	**―**	**―**	**―**	463	11	18
CP-04	661	607	47	54	611	18	50
CP-05	509	496	11	13	494	8	15
CP-06	294	**―**	**―**	**―**	277	9	17
CP-07	451	445	8	6	442	8	9
CP-08	544	520	18	24	540	4	4
CP-09	364	222	120	142	261	59	103
CP-10	506	329	176	177	425	60	81
CP-11	656	580	82	76	567	60	89
CP-12	423	406	27	17	413	12	10
CP-13	837	824	5	12	828	1	9
CP-14	472	278	199	194	396	33	75
CP-15	630	620	3	10	602	16	28
CP-16	355	332	28	23	349	5	6
CP-17	362	**―**	**―**	**―**	311	55	51
CP-18	601	**―**	**―**	**―**	597	2	4
CP-19	484	445	48	39	433	40	51
CP-20	391	390	1	1	390	3	1
CP-21	247	244	3	3	245	2	2
CP-22	610	528	67	82	458	76	152
CP-23	235	197	28	38	235	0	0
CP-24	*(1036)*	**―**	**―**	**―**	**―**	**―**	**―**
CP-25	602	**―**	**―**	**―**	337	157	265
CP-26	389	371	10	18	371	8	18
CP-27	130	130	0	0	129	1	1
CP-28	238	232	5	6	236	3	2
CP-29	290	**―**	**―**	**―**	172	20	118
CP-30	523	508	2	15	509	3	14
CP-31	*(948)*	**―**	**―**	**―**	**―**	**―**	**―**
CP-32	527	353	133	174	387	106	140
CP-33	449	432	24	17	437	50	12
CP-34	462	452	8	10	448	7	14
CP-35	484	**―**	**―**	**―**	345	138	139
CP-36	386	268	109	118	186	223	200
CP-37	342	190	145	152	337	6	5
CP-38	406	356	64	50	263	76	143
CP-39	518	510	8	8	476	39	42
CP-40	509	246	259	263	394	19	115
CP-41	349	341	3	8	348	1	1
CP-42	346	272	68	74	319	24	27
CP-43	460	346	132	114	255	153	205
CP-44	321	318	2	3	317	4	4
CP-45	357	326	17	31	283	76	74
CP-46	499	**―**	**―**	**―**	465	24	34
CP-47	537	505	17	32	408	49	129
CP-48	637	560	66	77	434	154	203
CP-49	451	399	58	52	385	40	66
CP-50	781	**―**	**―**	**―**	649	30	132
CP-51	621	**―**	**―**	**―**	569	52	52
CP-52	728	494	38	234	440	54	288
CP-53	562	561	0	1	448	49	114
CP-54	397	**―**	**―**	**―**	329	37	68
CP-55	793	339	263	454	**―**	**―**	**―**
CP-56	742	533	160	206	623	6	119
CP-57	507	501	5	6	503	4	4
CP-58	525	523	1	2	523	2	2
CP-59	405	405	1	0	403	2	2
CP-60	512	502	1	10	493	10	19
CP-61	397	396	7	1	395	2	2
CP-62	572	**―**	**―**	**―**	494	6	78
CP-63	610	608	0	2	609	0	1
CP-64	382	381	10	1	378	18	4
CP-65	369	365	1	4	364	5	5
CP-66	468	467	1	1	467	1	1
CP-67	539	**―**	**―**	**―**	479	35	60
CP-68	327	275	55	52	324	24	3
CP-69	587	585	0	2	583	2	4
CP-70	422	376	36	46	366	63	56
UP-01	239	190	51	49	152	80	87
UP-02	286	**―**	**―**	**―**	197	71	89
UP-03	264	**―**	**―**	**―**	183	35	81
UP-04	258	244	7	14	217	30	41
UP-05	462	**―**	**―**	**―**	288	124	174
UP-06	458	371	29	87	382	16	76
UP-07	376	370	3	6	368	4	8
UP-08	257	255	1	2	255	2	2
UP-09	286	278	0	8	276	9	10
UP-10	165	151	6	14	148	13	17
UP-11	417	343	71	74	351	35	66
UP-12	339	260	87	79	309	27	30
UP-13	106	92	1	14	97	7	9
UP-14	340	333	5	7	331	10	9
UP-15	214	189	0	25	214	1	0
UP-16	221	195	19	26	180	40	40
UP-17	613	596	7	17	481	63	132
UP-18	350	348	1	2	347	4	3
UP-19	116	**―**	**―**	**―**	60	56	56
UP-20	617	613	2	4	436	58	181
UP-21	305	303	2	2	303	5	2
UP-22	*(700)*	**―**	**―**	**―**	**―**	**―**	**―**
UP-23	565	565	2	0	562	8	3
UP-24	349	329	11	20	324	11	25
UP-25	244	**―**	**―**	**―**	166	56	78
UP-26	160	**―**	**―**	**―**	157	1	3
UP-27	269	246	37	23	180	96	89
UP-28	NA	NA	NA	NA	NA	NA	NA
UP-29	146	141	1	5	140	6	6
UP-30	228	228	16	0	189	41	39
**Total**	**41,320**	**29,583**	**2976**	**3678**	**35,383**	**3182**	**5142**

## References

[1] Hieronymi U., Kramme R., Kramme R., Hoffmann K.P., Pozos R.S. (2011). Cardiovascular Monitoring. Springer Handbook of Medical Technology.

[2] Perret-Guillaume C., Joly L., Benetos A. (2009). Heart Rate as a Risk Factor for Cardiovascular Disease. Prog. Cardiovasc. Dis..

[3] Polley C., Jayarathna T., Gunawardana U., Naik G., Hamilton T., Andreozzi E., Bifulco P., Esposito D., Centracchio J., Gargiulo G. (2021). Wearable Bluetooth Triage Healthcare Monitoring System. Sensors.

[4] Galli A., Montree R.J.H., Que S., Peri E., Vullings R. (2022). An Overview of the Sensors for Heart Rate Monitoring Used in Extramural Applications. Sensors.

[5] Berbari E.J., Bronzino J.D. (1999). Principles of electrocardiography. Biomedical Engineering Handbook.

[6] Nazeran H., Webster J.G. (2006). Electrocardiography, Computers in. Encyclopedia of Medical Devices and Instrumentation.

[7] Clark J.W.J., Webster J.G. (2010). The Origin of Biopotentials. Medical Instrumentation: Application and Design.

[8] Vieau S., Iaizzo P.A., Iaizzo P.A. (2015). Basic ECG Theory, 12-Lead Recordings and Their Interpretation. Handbook of Cardiac Anatomy, Physiology and Devices.

[9] Sattar Y., Chhabra L. (2023). Electrocardiogram.

[10] Luz E.J., Schwartz W.R., Cámara-Chávez G., Menotti D. (2016). ECG-based heartbeat classification for arrhythmia detection: A survey. Comput. Methods Programs Biomed..

[11] Landreani F., Caiani E.G. (2017). Smartphone accelerometers for the detection of heart rate. Expert Rev. Med. Devices.

[12] De Pinho Ferreira N., Gehin C., Massot B. (2021). A Review of Methods for Non-Invasive Heart Rate Measurement on Wrist. IRBM.

[13] Allen J. (2007). Photoplethysmography and its application in clinical physiological measurement. Physiol. Meas..

[14] Pereira T., Tran N., Gadhoumi K., Pelter M.M., Do D.H., Lee R.J., Colorado R., Meisel K., Hu X. (2020). Photoplethysmography based atrial fibrillation detection: A review. NPJ Digit. Med..

[15] Biswas D., Simões-Capela N., Van Hoof C., Van Helleputte N. (2019). Heart Rate Estimation From Wrist-Worn Photoplethysmography: A Review. IEEE Sens. J..

[16] Andreozzi E., Sabbadini R., Centracchio J., Bifulco P., Irace A., Breglio G., Riccio M. (2022). Multimodal Finger Pulse Wave Sensing: Comparison of Forcecardiography and Photoplethysmography Sensors. Sensors.

[17] Fine J., Branan K.L., Rodriguez A.J., Boonya-ananta T., Ajmal, Ramella-Roman J.C., McShane M.J., Coté G.L. (2021). Sources of Inaccuracy in Photoplethysmography for Continuous Cardiovascular Monitoring. Biosensors.

[18] Ismail S., Akram U., Siddiqi I. (2021). Heart rate tracking in photoplethysmography signals affected by motion artifacts: A review. EURASIP J. Adv. Signal. Process..

[19] Hajar R. (2018). The Pulse in Ancient Medicine Part 1. Heart Views.

[20] Luisada A.A., Singhal A., Portaluppi F. (1985). Assessment of Left Ventricular Function by Noninvasive Methods. Adv. Cardiol..

[21] Gordon J.W. (1877). On certain molar movements of the human body produced by the circulation of blood. J. Anat. Physiol..

[22] Burger H.C., Noordergraaf A. (1956). Physical basis of ballistocardiography. III. Am. Heart J..

[23] Starr I. (1958). The relation of the ballistocardiogram to cardiac function. Am. J. Cardiol..

[24] Knoop A.A. (1965). Experimental investigations on ultra-low frequency displacement ballistocardiography: NASA TT F-269. NASA Contractor Report—NASA CR.

[25] Sadek I., Biswas J., Abdulrazak B. (2019). Ballistocardiogram signal processing: A review. Health Inf. Sci. Syst..

[26] Inan O.T. Recent advances in cardiovascular monitoring using ballistocardiography. Proceedings of the Annual International Conference of the IEEE Engineering in Medicine and Biology Society (EMBC).

[27] Zanetti J., Salerno D. (1990). Seismocardiography: A new technique for recording cardiac vibrations: Concept, method, and initial observations. J. Cardiovasc. Technol..

[28] Inan O.T., Migeotte P.F., Park K.S., Etemadi M., Tavakolian K., Casanella R., Zanetti J., Tank J., Funtova I., Prisk G.K. (2015). Ballistocardiography and Seismocardiography: A Review of Recent Advances. IEEE J. Biomed. Health Inform..

[29] Taebi A., Solar B.E., Bomar A.J., Sandler R.H., Mansy H.A. (2019). Recent Advances in Seismocardiography. Vibration.

[30] Zanetti J.M., Tavakolian K. Seismocardiography: Past, present and future. Proceedings of the Annual International Conference of the IEEE Engineering in Medicine and Biology Society (EMBC).

[31] Crow R.S., Hannan P., Jacobs D., Hedquist L., Salerno D.M. (1994). Relationship between Seismocardiogram and Echocardiogram for Events in the Cardiac Cycle. Am. J. Noninvas. Cardiol..

[32] Khosrow-Khavar F., Tavakolian K., Blaber A., Menon C. (2017). Automatic and Robust Delineation of the Fiducial Points of the Seismocardiogram Signal for Noninvasive Estimation of Cardiac Time Intervals. IEEE Trans. Biomed. Eng..

[33] Di Rienzo M., Vaini E., Castiglioni P., Merati G., Meriggi P., Parati G., Faini A., Rizzo F. (2013). Wearable seismocardiography: Towards a beat-by-beat assessment of cardiac mechanics in ambulant subjects. Auton. Neurosci..

[34] Rappaport M.B., Sprague H.B. (1942). The graphic registration of the normal heart sounds. Am. Heart J..

[35] Dimond E.G., Benchimol A. (1961). Phonocardiography. Calif. Med..

[36] Ismail S., Siddiqi I., Akram U. (2018). Localization and classification of heart beats in phonocardiography signals—A comprehensive review. EURASIP J. Adv. Signal. Process..

[37] Giordano N., Knaflitz M. (2019). A Novel Method for Measuring the Timing of Heart Sound Components through Digital Phonocardiography. Sensors.

[38] Greenstein J. (1955). Phonocardiography; its application to clinical medicine. S. Afr. Med. J..

[39] Vermarien H., Webster J.G. (2006). Phonocardiography. Encyclopedia of Medical Devices and Instrumentation.

[40] Andreozzi E., Fratini A., Esposito D., Naik G., Polley C., Gargiulo G.D., Bifulco P. (2020). Forcecardiography: A Novel Technique to Measure Heart Mechanical Vibrations onto the Chest Wall. Sensors.

[41] Andreozzi E., Gargiulo G.D., Esposito D., Bifulco P. (2021). A Novel Broadband Forcecardiography Sensor for Simultaneous Monitoring of Respiration, Infrasonic Cardiac Vibrations and Heart Sounds. Front. Physiol..

[42] Andreozzi E., Centracchio J., Punzo V., Esposito D., Polley C., Gargiulo G.D., Bifulco P. (2021). Respiration Monitoring via Forcecardiography Sensors. Sensors.

[43] Andreozzi E., Centracchio J., Esposito D., Bifulco P. (2022). A Comparison of Heart Pulsations Provided by Forcecardiography and Double Integration of Seismocardiogram. Bioengineering.

[44] Centracchio J., Andreozzi E., Esposito D., Gargiulo G.D. (2022). Respiratory-Induced Amplitude Modulation of Forcecardiography Signals. Bioengineering.

[45] Centracchio J., Andreozzi E., Esposito D., Gargiulo G.D., Bifulco P. (2022). Detection of Aortic Valve Opening and Estimation of Pre-Ejection Period in Forcecardiography Recordings. Bioengineering.

[46] Centracchio J., Esposito D., Gargiulo G.D., Andreozzi E. (2022). Changes in Forcecardiography Heartbeat Morphology Induced by Cardio-Respiratory Interactions. Sensors.

[47] Jafari Tadi M., Lehtonen E., Pankaala M., Saraste A., Vasankari T., Teras M., Koivisto T. Gyrocardiography: A new non-invasive approach in the study of mechanical motions of the heart. Concept, method and initial observations. Proceedings of the Annual International Conference of the IEEE Engineering in Medicine and Biology Society (EMBC).

[48] Jafari Tadi M., Lehtonen E., Saraste A., Tuominen J., Koskinen J., Teräs M., Airaksinen J., Pänkäälä M., Koivisto T. (2017). Gyrocardiography: A New Non-invasive Monitoring Method for the Assessment of Cardiac Mechanics and the Estimation of Hemodynamic Variables. Sci. Rep..

[49] Sieciński S., Kostka P.S., Tkacz E.J. (2020). Gyrocardiography: A Review of the Definition, History, Waveform Description and Applications. Sensors.

[50] Dehkordi P., Tavakolian K., Tadi M.J., Zakeri V., Khosrow-Khavar F. (2020). Investigating the estimation of cardiac time intervals using gyrocardiography. Physiol. Meas..

[51] D’Mello Y., Skoric J., Xu S., Roche P.J.R., Lortie M., Gagnon S., Plant D.V. (2019). Real-Time Cardiac Beat Detection and Heart Rate Monitoring from Combined Seismocardiography and Gyrocardiography. Sensors.

[52] Hossein A., Mirica D.C., Rabineau J., Del Rio J.I.J., Morra S., Gorlier D., Nonclercq A., van de Borne P., Migeotte P.F. (2019). Accurate Detection of Dobutamine-induced Haemodynamic Changes by Kino-Cardiography: A Randomised Double-Blind Placebo-Controlled Validation Study. Sci. Rep..

[53] Hossein A., Rabineau J., Gorlier D., Del Rio J.I.J., van de Borne P., Migeotte P.F., Nonclercq A. (2021). Kinocardiography Derived from Ballistocardiography and Seismocardiography Shows High Repeatability in Healthy Subjects. Sensors.

[54] Tendulkar A.P., Harken A.H. (2006). Mechanics of the normal heart. J. Card Surg..

[55] Buckberg G., Hoffman J.I., Mahajan A., Saleh S., Coghlan C. (2008). Cardiac mechanics revisited: The relationship of cardiac architecture to ventricular function. Circulation.

[56] Bloechlinger S., Grander W., Bryner J., Dünser M.W. (2011). Left ventricular rotation: A neglected aspect of the cardiac cycle. Intensive Care Med..

[57] Yang C., Tavassolian N. A feasibility study on a low-cost, smartphone-based solution of pulse transit time measurement using cardio-mechanical signals. Proceedings of the IEEE Healthcare Innovations and Point of Care Technologies (HI-POCT).

[58] Sieciński S., Kostka P.S., Tkacz E.J. (2020). Heart Rate Variability Analysis on Electrocardiograms, Seismocardiograms and Gyrocardiograms on Healthy Volunteers. Sensors.

[59] Siecinski S., Kostka P.S., Tkacz E.J. Time Domain And Frequency Domain Heart Rate Variability Analysis on Gyrocardiograms. Proceedings of the Annual International Conference of the IEEE Engineering in Medicine & Biology Society (EMBC).

[60] Lahdenoja O., Hurnanen T., Iftikhar Z., Nieminen S., Knuutila T., Saraste A., Kiviniemi T., Vasankari T., Airaksinen J., Pänkäälä M. (2018). Atrial Fibrillation Detection via Accelerometer and Gyroscope of a Smartphone. IEEE J. Biomed. Health Inform..

[61] Iftikhar Z., Lahdenoja O., Jafari Tadi M., Hurnanen T., Vasankari T., Kiviniemi T., Airaksinen J., Koivisto T., Pänkäälä M. (2018). Multiclass Classifier based Cardiovascular Condition Detection Using Smartphone Mechanocardiography. Sci. Rep..

[62] Hurnanen T., Kaisti M., Jafari Tadi M., Vähä-Heikkilä M., Nieminen S., Iftikhar Z., Paukkunen M., Pänkäälä M., Koivisto T. Heartbeat Detection Using Multidimensional Cardiac Motion Signals and Dynamic Balancing. Proceedings of the European Medical and Biological Engineering Confernce (EMBEC) & Nordic-Baltic Conference on Biomedical Engineering and Medical Physics (NBC).

[63] Jafari Tadi M., Lehtonen E., Teuho J., Saraste A., Pänkäälä M., Teräs M., Koivisto T. A miniaturized MEMS motion processing system for nuclear medicine imaging applications. Proceedings of the 2016 Computing in Cardiology Conference (CinC).

[64] Jafari Tadi M., Teuho J., Lehtonen E., Saraste A., Pänkäälä M., Koivisto T., Teräs M. (2017). A novel dual gating approach using joint inertial sensors: Implications for cardiac PET imaging. Phys. Med. Biol..

[65] Jafari Tadi M., Lehtonen E., Teuho J., Koskinen J., Schultz J., Siekkinen R., Koivisto T., Pänkäälä M., Teräs M., Klén R. (2019). A Computational Framework for Data Fusion in MEMS-Based Cardiac and Respiratory Gating. Sensors.

[66] Hernandez J.E., Cretu E. Simple Heart Rate Monitoring System with a MEMS Gyroscope for Sleep Studies. Proceedings of the Annual Information Technology, Electronics and Mobile Communication Conference (IEMCON).

[67] Kaisti M., Jafari Tadi M., Lahdenoja O., Hurnanen T., Saraste A., Pänkäälä M., Koivisto T. (2019). Stand-Alone Heartbeat Detection in Multidimensional Mechanocardiograms. IEEE Sens. J..

[68] Jia W., Li Y., Bai Y., Mao Z.-H., Sun M., Zhao Q. Estimation of heart rate from a chest-worn inertial measurement unit. Proceedings of the 2015 International Symposium on Bioelectronics and Bioinformatics (ISBB).

[69] Lee H., Lee H., Whang M. (2018). An Enhanced Method to Estimate Heart Rate from Seismocardiography via Ensemble Averaging of Body Movements at Six Degrees of Freedom. Sensors.

[70] Aboulezz E., Skoric J., D’Mello Y., Hakim S., Clairmonte N., Lortie M., Plant D.V. Analyzing Heart Rate Estimation from Vibrational Cardiography with Different Orientations. Proceedings of the Annual International Conference of the IEEE Engineering in Medicine and Biology Society (EMBC).

[71] Lahdenoja O., Humanen T., Jafari Tadi M., Pänkäälä M., Koivisto T. Heart rate variability estimation with joint accelerometer and gyroscope sensing. Proceedings of the 2016 Computing in Cardiology Conference (CinC).

[72] Hu Y., Wong Y., Wei W., Du Y., Kankanhalli M., Geng W. (2018). A novel attention-based hybrid CNN-RNN architecture for sEMG-based gesture recognition. PLoS ONE.

[73] Xia Y., Wulan N., Wang K., Zhang H. (2018). Detecting atrial fibrillation by deep convolutional neural networks. Comput. Biol. Med..

[74] Jia Z., Ji J., Zhou X., Zhou Y. (2022). Hybrid spiking neural network for sleep electroencephalogram signals. Sci. China Inf. Sci..

[75] Duraj K.M., Siecinski S., Doniec R.J., Piaseczna N.J., Kostka P.S., Tkacz E.J. Heartbeat detection in seismocardiograms with semantic segmentation. Proceedings of the 44th Annual International Conference of the IEEE Engineering in Medicine and Biology Society (EMBC).

[76] Zhang S., Zhang H., Lin Z., Huat Ng S. (2022). Automated and precise heartbeat detection in ballistocardiography signals using bidirectional LSTM. Franklin Open.

[77] Yoo J.C., Han T.H. (2009). Fast Normalized Cross-Correlation. Circuits Syst. Signal Process.

[78] Briechle K., Hanebeck U.D. Template matching using fast normalized cross correlation. Proceedings of the Society of Photo-Optical Instrumentation Engineers (SPIE) 4387, Optical Pattern Recognition XII.

[79] Chen Y.H., Chen H.H., Chen T.C., Chen L.G. Robust heart rate measurement with phonocardiogram by on-line template extraction and matching. Proceedings of the Annual International Conference of the IEEE Engineering in Medicine and Biology Society (EMBC).

[80] Centracchio J., Parlato S., Esposito D., Bifulco P., Andreozzi E. (2023). ECG-Free Heartbeat Detection in Seismocardiography Signals via Template Matching. Sensors.

[81] Yang C., Fan F., Aranoff N., Green P., Li Y., Liu C., Tavassolian N. (2021). An Open-Access Database for the Evaluation of Cardio-Mechanical Signals From Patients with Valvular Heart Diseases. Front. Physiol..

[82] Pan J., Tompkins W.J. (1985). A real-time QRS detection algorithm. IEEE Trans. Biomed. Eng..

[83] Sedghamiz H. (2018). BioSigKit: A Matlab Toolbox and Interface for Analysis of BioSignals. J. Open. Source Softw..

[84] Altman D.G., Bland J.M. (1983). Measurement in medicine: The analysis of method comparison studies. Statistician.

[85] Giavarina D. (2015). Understanding Bland Altman analysis. Biochem. Med..

[86] Ran K. (2020). Bland-Altman and Correlation Plot, MATLAB Central File Exchange. https://www.mathworks.com/matlabcentral/fileexchange/45049-bland-altman-and-correlation-plot.

[87] Işilay Zeybek Z.M., Racca V., Pezzano A., Tavanelli M., Di Rienzo M. (2022). Can Seismocardiogram Fiducial Points Be Used for the Routine Estimation of Cardiac Time Intervals in Cardiac Patients?. Front. Physiol..

